# DNA Nanotechnology Enters Cell Membranes

**DOI:** 10.1002/advs.201900043

**Published:** 2019-03-20

**Authors:** Shuaidong Huo, Hongyan Li, Arnold J. Boersma, Andreas Herrmann

**Affiliations:** ^1^ DWI‐Leibniz Institute for Interactive Materials Forckenbeckstr. 50 52056 Aachen Germany; ^2^ Zernike Institute for Advanced Materials University of Groningen Nijenborgh 4 9747 AG Groningen The Netherlands; ^3^ Institute of Technical and Macromolecular Chemistry RWTH Aachen University Worringerweg 2 52074 Aachen Germany

**Keywords:** DNA amphiphiles, membranes, nanopores, nanostructures, vesicles

## Abstract

DNA is more than a carrier of genetic information: It is a highly versatile structural motif for the assembly of nanostructures, giving rise to a wide range of functionalities. In this regard, the structure programmability is the main advantage of DNA over peptides, proteins, and small molecules. DNA amphiphiles, in which DNA is covalently bound to synthetic hydrophobic moieties, allow interactions of DNA nanostructures with artificial lipid bilayers and cell membranes. These structures have seen rapid growth with great potential for medical applications. In this Review, the current state of the art of the synthesis of DNA amphiphiles and their assembly into nanostructures are first summarized. Next, an overview on the interaction of these DNA amphiphiles with membranes is provided, detailing on the driving forces and the stability of the interaction. Moreover, the interaction with cell surfaces in respect to therapeutics, biological sensing, and cell membrane engineering is highlighted. Finally, the challenges and an outlook on this promising class of DNA hybrid materials are discussed.

## 1 Introduction

Embedded in a unique language, deoxyribonucleic acid (DNA) carries the lion's share of the hereditary information in living cells. Ever since Friedrich Miescher isolated DNA in 1869,^1^ the scientific community extensively investigated its properties and possible applications. James Watson and Francis Crick identified the molecular structure of DNA in 1953,^2^ starting the age of genetics and modern molecular biology.

The Watson‐Crick base pairing rules provide DNA with unique self‐recognition and sequence programmability, which enabled DNA and DNA‐based materials to find their applications in biomedicine, which includes drug delivery, gene silencing, and diagnostics. Apart from that technologies have been developed to evolve DNA molecules, which strongly bind a wide variety of target molecules (aptamers) or exhibit catalytic activity (DNAzymes).^3^, ^4^, ^5^, ^6^ As therapeutics, nucleic acids inhibit either DNA or RNA expression, thereby blocking the production of proteins related to a disease.^7^ However, the clinical application of therapeutic nucleic acids (TNAs) is still facing limitations due to unsolved challenges regarding delivery. For instance, negatively charged cellular membranes act as a natural barrier to prevent entry of foreign polyanionic nucleic acids. Once inside the cell, DNases or RNases degrade foreign nucleic acids to prevent their integration into the genome.^8^ TNAs further have to be delivered to the correct cells with minimal side effects to other cells.^9^ When using TNAs as artificial receptors, the failed anchoring or insertion of the DNA in the cell membrane restricts its excellent recognition properties. These challenges potentially decrease the applicability of DNA reporting signals from the cell or tissue.

The unique programmability gives DNA an edge over other molecules that interact with membranes, such as peptides, proteins, and small molecules. In order to realize successful insertion of DNA in the cell membrane and efficient delivery of TNAs both in vitro and in vivo, one of the most commonly used strategies is increasing the hydrophobicity of nucleic acids. To this end, DNA is chemically conjugated with hydrophobic moieties, resulting in DNA amphiphiles. Efficient and stable insertion into live cell membranes allows amphiphilic DNA conjugates to cross the cell membrane.^10^, ^11^, ^12^, ^13^ Importantly, these DNA amphiphiles can be modified with additional functional groups that enable specific targeting and biocompatibility in vivo, providing them with a tremendous potential for biomedicine.^14^, ^15^, ^16^, ^17^

To date, the synthesis and application of amphiphilic DNA conjugates have been well demonstrated and reviewed.^18^, ^19^, ^20^

## 2 Synthesis of DNA Amphiphiles

A DNA amphiphile is based on hydrophilic DNA that contains a covalently connected hydrophobic segment.^19^ Usually, the hydrophobic moiety is a polymer or a small molecule. The lipophilic modifications of DNA can be achieved by conjugation at either the 3′‐ or 5′‐terminal, or within the DNA sequence, allowing the construction of complex structures.^21^, ^22^, ^23^, ^24^

These hydrophobic moieties can be conjugated to DNA, either on a solid support during DNA synthesis or by coupling to already synthesized DNA units in solution. The first successful chemical synthesis of a dinucleotide was achieved in 1955.^25^ Stable deoxynucleoside phosphoramidites were introduced as synthons in 1985, opening up the field.^26^ Nowadays, solid phase synthesis (SPS) allows generating DNA fragments of up to 200 nucleotides. This technology allows functionalization or introduction of non‐natural nucleotides.^27^ The fully automated synthesis can be precisely controlled, monitored, and is characterized by a high reproducibility. To broaden the scope of synthesis robots by introducing special solvents, catalysts, extreme reaction conditions or long reaction times, the automated process can be replaced by the syringe synthesis technique or in‐flask reactions to realize various modifications of the DNA with hydrophobic units.^20^

Coupling of DNA with specific motifs in solution phase has been demonstrated as another highly versatile strategy, which was reviewed by our group before.^19^ Solution phase synthesis is used for covalent bond formation between functional groups such as amines^28^ or thiols,^29^ with groups such as carboxylic acids^30^ or maleimides.^31^ However, aqueous solution coupling of DNA with hydrophobic molecules often results in low yields due to the solvent incompatibility of starting materials. To overcome this limitation, we reported a conjugation protocol for coupling of hydrophobic molecules to DNA with high efficiency.^32^ By complexing DNA with positively charged quaternary ammonium surfactants, we neutralized the charge on the DNA, making it soluble in organic solvent. The organic phase coupling technique expands the number of possibilities to generate amphiphilic DNA hybrids.

**Figure 1:**
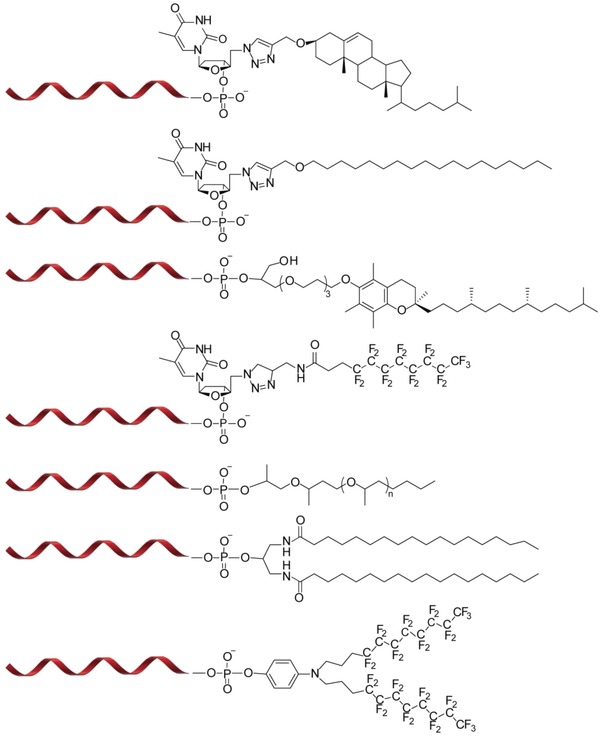
Selected lipid‐oligonucleotide conjugates, exemplifying the variety of lipophilic residues that can be appended to DNA. Structures of DNA conjugated with, from top to bottom, cholesterol obtained via a 1,3‐dipolar Huisgen's cycloaddition between alkyne modified cholesterol and 5′‐azido‐5′‐deoxythymidine^39^; a single hydrocarbon chain obtained via a 1,3‐dipolar Huisgen's reaction between alkyne modified C18 chain and 5′‐azido‐5′‐deoxythymidine^39^; tocopherol obtained by covalent attachment to the 5′ end of the strand^40^, ^41^; a single fluorocarbon chain obtained via a Huisgen's reaction between 5′‐azide deoxythymidine and propargylated fluorocarbon chain^42^; a PPO chain obtained via a PPO phosphoramidite during SPS^21^; double hydrocarbon chains obtained via a reaction of stearoyl chloride with 1,3‐diamino‐2‐dydroxypropane^40^, ^43^; and double fluorocarbon chains obtained by a diperfluorodecyl phosphoramidite during SPS.^44^

One of the most commonly used lipids in DNA amphiphiles is cholesterol. In addition to cholesterol or one of its derivatives, other synthetic single‐chain fatty acids,^33^ steroid molecules,^34^ α‐tocopherol,^35^ hydrophobic polymers, such as poly(propylene oxide) (PPO),^21^ or the π‐conjugated system porphyrin^36^, ^37^ have been successfully introduced to DNA (**Figure** **1**). Hence, synthetic protocols to introduce a wide range of hydrophobic moieties into DNA at various positions are available, allowing for the exploration of new functionalities in nanotechnology.^38^

## 3 Nanoscale Assemblies from DNA Amphiphiles

DNA amphiphiles can be designed to assemble into a variety of nanoscale structures. In general, nanoscale structures can be constructed “top‐down” or “bottom‐up”: The bottom‐up approach makes use of assembling single molecules into nanostructures by intermolecular interactions, yielding a level of molecular control that is out of reach to a top‐down strategy.

DNA amphiphiles that contain both hydrophobic moieties and nucleic acids possess advantageous features derived from the DNA part as well as from the hydrophobic moieties combined in one molecule. The Watson‐Crick base pairing rules that govern DNA nanotechnology allow the rational design of complex nanostructures which result in novel functions. This molecular technology is based on bottom‐up self‐assembly, which was initiated by Nadrian Seeman in the early 1980s and has been growing rapidly ever since.^45^ Depending on the design, the structures can be 1D, 2D, or 3D. In addition, single‐stranded overhanging sequences in the final structure enable further functionalization by hybridization with complementary sequences. More detail on the assembly of DNA nanostructures and their emerging applications in areas such as biophysics, drug delivery, synthetic biology, can be found in ref. 41, 46.

**Figure 2:**
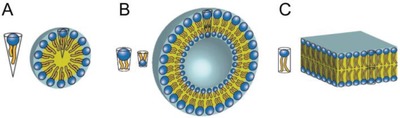
Schematic models of self‐assembled lipids. A) Micelles are preferentially formed by lipids with a conical shape. B) Vesicles are composed of spherical lipid bilayers with a water core. C) Planar lipid bilayers are formed by lipids with a cylindrical shape. Reproduced with permission.^52^ Copyright 2014, American Chemical Society.

On the other hand, hydrophobic units in amphiphiles tend to microphase separate due to hydrophobic interactions.^47^, ^48^, ^49^ This structural concept can be further combined with assembly mechanisms relying on electrostatic forces,^50^ π–π stacking interactions,^51^ hydrogen bonding and Van der Waals interactions. Hence, DNA amphiphiles have the ability to self‐assemble into predictable morphologies (**Figure** **2**), such as spherical micelles, rods, vesicles, and bilayers.^52^ An inspiring example of engineering such morphologies was reported by Baglioni and co‐workers in 2007^53^: They synthesized nucleolipids in which the choline headgroup of phosphatidylcholines was replaced by a nucleoside, either uridine or adenosine. The resulting mole‐cules had a negatively charged nucleotide group as polar head. Depending on the length of the alkyl chains, globular micelles, flexible cylindrical aggregates, or bilayers were obtained from these nucleolipids. The shape of the amphiphile dictates the obtained structures: a short hydrophobic chain provides an amphiphile with a conical shape, resulting in globular micellar aggregates, while a long alkyl chain gives a cylindrical shape that results in wormlike micellar aggregates. The latter morphology is further modulated by improved orientation of the bases that interact with each other.

### 3.1 Micelles from DNA Amphiphiles

When above its critical micelle concentration, DNA amphiphiles self‐assemble into micellar systems with nanometer dimensions.^54^ This occurs spontaneously because the amphiphiles phase separate in aqueous media. Micellar structures are composed of a hydrophobic core and a hydrophilic DNA shell.

#### 3.1.1 Formation and Structure of DNA Amphiphile Micelles

**Figure 3:**
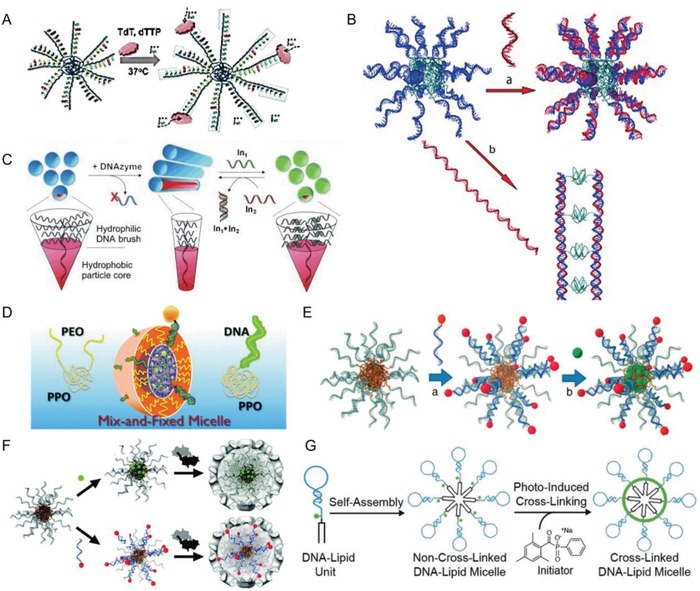
A) Enzymatic growth of DNA‐*b*‐PPO micelles. Reproduced from ref. 59. B) Schematic representation of hybridization of DNA‐*b*‐PPO micelles with different DNA molecules. a) Base pairing with a short complementary sequence yields micelles and maintains the overall shape of the aggregates. b) Hybridization with long DNA templates results in rod‐like micelles. Reproduced from ref. 61. C) DNAzyme induced reversible transformation of the aggregate shape of a DNA‐brush block copolymer. Reproduced from ref. 62. D) Schematic of the mixed micelle architecture. Two amphiphilic block copolymers, DNA‐*b*‐PPO and PEO‐*b*‐PPO‐*b*‐PEO with the trade name Pluronic F127, form mixed micellar structure and this micelle can be stabilized by formation of a semi‐interpenetrating network in its core. Reproduced with permission.^63^ Copyright 2010, Royal Society of Chemistry. E) Schematic representation of the drug delivery system based on DNA amphiphiles. a) Targeting units (red dots) that are connected to the complementary sequence of the micelles are hybridized to equip the nanoparticle surface with folic acid units. b) The anticancer drug (green dots) is loaded into the core of the micelles. Reproduced from ref. 55. F) Schematic of DNA micelle‐templated VC formation. Loading hydrophobic molecules (top, green) into micelle core and hybridization of a complementary DNA connected to functional moieties (bottom, red) to the DNA micelle. Reproduced with permission.^66^ Copyright 2010, American Chemical Society. G) Photoinduced cross‐linking of self‐assembled DNA‐methacrylamide‐lipid micelles. Green dot between DNA and lipid represents methacrylamide molecules which can be crosslinked. Reproduced from ref. 65.

DNA amphiphiles form spherical micelles with a diameter from 6.7 to 36.4 nm, as measured by atomic force microscopy (AFM) and dynamic light scattering (DLS).^33^, ^54^, ^55^ Similar to inorganic nanoparticles,^56^, ^57^, ^58^ the size of the spherical micelles can be regulated by adjusting the DNA or hydrophobic segments. AFM revealed that such micelles deform, depending on the hydrophobic segments attached to the DNA molecules. Amphiphiles with different DNA lengths or different lipids form micelles with tunable size, indicating a relationship between micelle size and length of the constituent segments. In this context, DNA polymerase can be utilized to control the size of micelles: Treatment of micelles consisting of DNA‐*b*‐PPO (PPO block covalently connected to the 5‐end of a 22 nt single‐stranded DNA) with the enzyme terminal deoxynucleotidyl transferase (TdT) increases the size from 10 to 23 nm, depending on the incubation time (**Figure** **3**A).^59^ Similarly, the use of enzymes to digest and ligate nucleic acids resulted in DNA amphiphiles containing dsDNA with molecular weights of up to three million Daltons.^60^ These strategies offer post‐synthetic control over the growth of DNA nanostructures in aqueous medium. Furthermore, the size and stability of DNA amphiphile micelles is determined by the number of hydrophobic moieties: Increasing the number of nucleotides containing dodec‐1‐ynyl chains attached to the nucleobases resulted in smaller micelles with increased stability. The position of the hydrophobic nucleotide units in the short sequences proved to have little influence on micelle structure and stability.^33^

Hybridization allows precise post‐synthetic control over the shape of a DNA micelle (Figure 3B). The shape of micelles can be changed from spheres to rods by addition of complementary single‐stranded DNA to the DNA amphiphiles, forming double‐stranded DNA.^61^ Morphology can be controlled reversibly with for example DNA‐brush amphiphiles that assemble into spherical micelles (≈25 nm) and contain a RNA nucleotide as an enzymatic cleavage site (Figure 3C).^62^ Mixing spherical micelles with a DNA‐based phosphodiesterase that is specific for the DNA sequence and cuts at the RNA site, resulted in a long cylindrical structure (>1000 nm in length). To facilitate a subsequent cylinder‐to‐sphere transformation, a 19‐base ssDNA sequence was added, which forms a 9 nt duplex with the truncated DNA of the cylinder shell. The reverse sphere‐to‐cylinder transition was achieved again by the addition of a complementary 19‐base ssDNA designed to invade into the shorter nine‐base duplex in the micelle shell. Thus, DNA is a superb tool for encoding supramolecular structure information allowing exquisite control over morphology of DNA amphiphiles.

Our group synthesized an additional type of structure, based on a mixed hybrid micellar architecture (Figure 3D).^63^ Here, DNA‐*b*‐PPO and Pluronic F127 (a triblock copolymer with a PEG (polyethylene glycol)‐*b*‐PPO‐*b*‐PEG architecture) were combined. In this construct, the PPO from both DNA amphiphile and Pluronic copolymer formed the core of the micelles, while DNA from DNA‐*b*‐PPO and PEG from Pluronic were located in the corona. The resulting self‐assembled structures were finally cross‐linked by forming a semi interpenetrating polymer network in the micelle core. The PEG domain did not undermine the hybridization of DNA and the hydrophobic core could be loaded with hydrophobic drugs. The resulting aggregates exhibit the potential for combining block copolymers of different nature, facile functionalization of DNA amphiphiles by hybridization and the possibility for stabilization of such aggregates by polymer network formation within the micelle core. As a result, micelles were obtained that are stable in regard to dilution, temperature increase and the possibility for attaching conveniently targeting units. Likely, such a PEG corona shields the DNA backbone and improves the biocompatibility and immune compatibility of the mixed hybrid micelles,vide infra.

#### 3.1.2 Functionalization and Features of DNA Amphiphile Micelles

DNA amphiphile micelles can be functionalized to introduce new properties. Micellar aggregation of DNA amphiphiles aligns the single‐stranded DNA in its corona, which allows DNA‐templated organic reactions to proceed in 3D space. Therefore, the ssDNA of the corona needs to be hybridized with sequences, which are equipped with reactants.^55^ (Figure 3E)

Moreover, DNA amphiphiles were functionalized to a high degree for combined mRNA detection and gene therapy in molecular beacon micelle flares (MBMFs), which are self‐assembled diacyllipid‐molecular‐beacon DNA conjugates.^64^ The MBMFs showed efficient cell uptake, enhanced enzymatic stability, excellent target selectivity, and superior biocompatibility compared to pristine DNA. Diperfluorodecyl‐DNA conjugates allow further improvement of target binding affinity and enzymatic resistance by virtue of the physicochemical properties of fluorination.^44^ However, loss of integrity of micelles compromised the recognition ability of the aptamer when interacting with cells. Therefore, the same group developed a more stable cross‐linked DNA‐methacrylamide‐lipid micelle (X‐DLM) system (Figure 3F), which incorporates a methacrylamide functionality between the hydrophilic and hydrophobic portions of DNA‐lipid amphiphiles that can be cross‐linked after self‐assembly in aqueous solution.^65^ This X‐DLM system offers further improved stability in the cellular environment and better specificity regarding cell recognition.

Besides cross‐linking of DNA amphiphiles, these nano‐objects can be encapsulated via a facile self‐assembly process. Therefore, the nucleic acid micelles were incubated with virus capsid (VC) proteins (Figure 3G).^66^ In this approach, the negatively charged DNA particles induced capsid formation, allowing the entrapment of oligonucleotides as a constituent part of the micellar template. The preloading of entities in the core or by hybridization of micelles enables encapsulation of various small molecules inside VCs, which marked a significant step forward in chemical virology due to the flexibility of loading these protein nanocontainers with various payloads. Thus, DNA amphiphiles form micelles that are tunable, versatile, and allow realization of functions.

### 3.2 Liposomes from DNA Amphiphiles

Next to micelles, amphiphilic DNA molecules can be aligned to form liposomes or bilayers, similar as indicated for conventional surfactant molecules in Figure 2: Liposomes are flat bilayer sheets folded to form closed spherical objects, with the structure of the assembly determined by the conical shape of the DNA amphiphiles.

#### 3.2.1 Formation and Structure of DNA Amphiphile Liposomes

**Figure 4:**
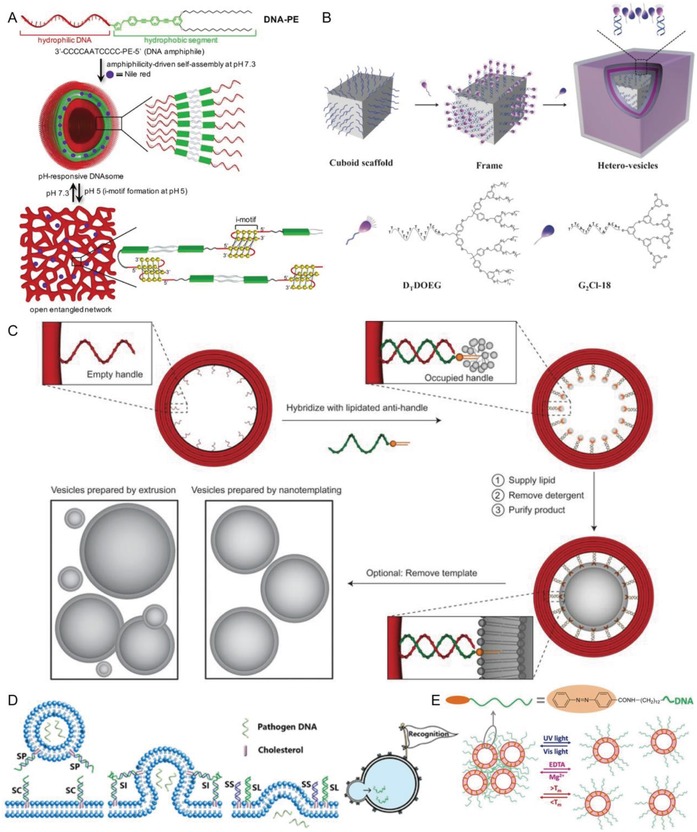
A) Illustration of working principle of reversible pH‐responsive DNAsome. At pH 7.3, C rich DNA‐PE spontaneously forms a DNAsome. When pH is lowered to 5, the i‐motif structure forms and the morphology of the DNAsome transforms to entangled 3D networks. Reproduced from ref. 68. B) Schematic of the frame‐guided assembly process with a DNA origami scaffold. DNA origami cuboid with A20 sequences protruding from the surface is folded by a template and corresponding staple strands. Then, D_T_DOEG dendron is anchored on DNA origami by hybridization. When G_2_Cl‐18 is added, hydrophobic groups on the DNA origami guide G_2_Cl‐18 dendrons to form hetero‐vesicles around the DNA frame. Reproduced from ref. 70. C) Size‐controlled liposome formation through a DNA scaffold. A DNA‐origami ring (red) with multiple single‐stranded empty handles is constructed first. Then DNA antihandles (oligonucleotides with complementary sequence to handle sequence that are chemically conjugated to DOPE, shown as green curl with orange head) are hybridized to the DNA ring. Afterward, this lipid‐modified ring is mixed with extra lipid and detergent, and dialysed to allow vesicle formation. After purification and release, uniform liposomes with sizes being determined by the DNA template are generated. Reproduced with permission.^71^ Copyright 2016, Nature Publishing Group. D) Schematic figure of pathogen DNA delivery to protocell by DNA‐mediated fusion. When anchoring a set of complementary DNA on a protocell and an artificial pathogen membrane, DNA hybridization brings the two membranes in close proximity to enable fusion. Thereby, pathogen DNA is released into the protocell. Reproduced with permission.^73^ Copyright 2018, American Chemical Society. E) Illustration of reversible control over the assembly of liposomes. When the liposome surface is equipped with self‐complementary DNA bearing a terminal azobenzene moiety, the vesicles undergo reversible assembly and disassembly in response to multiple stimuli including UV light, salt addition and temperature. Reproduced with permission.^78^ Copyright 2016, American Chemical Society.

Nucleic acid functionalization of lipids allows additional control over lipid self‐assembly through specific interactions among the polar heads. As in micelles, the hydrophobic lipid tail and hydrophilic DNA head combined determine the phase behavior and aggregate microstructure.^53^ DNA amphiphiles that form vesicular structures can be made for example by linking poly(butadiene) covalently to poly‐cytidine during solid phase synthesis.^67^ The resulting amphiphilic copolymer self‐assembled into 80 nm vesicles as demonstrated by TEM and confocal microscopy. By using a functional DNA moiety as head group, one can induce more complex behavior: Conjugation of the lipid tail with a DNA sequence that forms an i‐motif renders the liposome structure pH sensitive upon acidification (**Figure** **4**A).^68^ The C‐rich DNA segment undergoes a structural change from random coil ssDNA to an i‐motif structure upon acidification (pH = 5), triggering the transformation of the vesicles into an entangled 3D network. This process was reversed when the pH was increased to 7.3. This structure allowed the encapsulation of a hydrophobic molecule and a pH‐triggered release, showing that these DNA amphiphile systems can be engineered to be sensitive to external stimuli.

#### 3.2.2 Templated Vesicle Formation by DNA Amphiphile Assembly

Moreover, vesicles can be prepared with programmed geometry and dimensions using ssDNA‐modified gold nanoparticles or DNA origami as scaffolds.^69^, ^70^ The ssDNA on the scaffold hybridizes with corresponding DNA amphiphiles and the resulting frame allows generation of the desired bilayer upon mixing with additional DNA amphiphiles (Figure 4B). Strikingly, a variety of vesicle shapes was obtained by templating the DNA amphiphile assembly, i.e., cuboids and dumbbells. In a similar way, DNA origami can be used to template vesicle formation in the interior of the origami structure. This allows size‐controlled liposome formation with the added feature that the origami can be removed.^71^ In this case, the inner surface of the DNA origami ring is decorated with ssDNA extensions, which can hybridize with lipid‐DNA conjugates, thus acting as an exoskeleton for liposome formation (Figure 4C). Using this approach, a series of highly monodisperse sub‐100 nm (29, 46, 60, and 94 nm) liposomes with a variety of different lipid compositions were produced. Thus, DNA amphiphile vesicles with desired sizes or shapes can be synthesized using templated vesicle formation.

#### 3.2.3 Amphiphilic DNA Mediated Vesicle Fusion and Assembly

Besides exclusively preparing vesicles from DNA amphiphiles, liposomes formed from other lipids can be functionalized by nucleic acids with the help of amphiphilic DNA conju‐gates. Thereby, the hydrophobic unit of the DNA amphiphile pierces into the lipid membrane. In this context, DNA amphiphiles are excellent tools for controlled vesicle fusion and formation of multivesicle assemblies.^72^ For vesicle fusion, bilayers are brought into close proximity after which the lipid head‐groups from one vesicle insert into the other, creating the basis for the fusion pore. DNA hybridization connects vesicles and brings them together to initiate fusion. Using vesicles modified with double cholesterol terminated DNA strands that were complementary to each other, Höök and co‐workers reported for the first time amphiphilic DNA induced fusion of lipid vesicles.^73^ The hybridization occurs in a zipper‐like fashion by forcing the vesicles into close contact, enabling opening of the fusion pore between the two vesicles. DNA‐induced fusion was more efficient with liposomes that consisted of cone shaped lipids such as DOPE (1,2‐dioleyl‐*sn*‐glycero‐3‐phospho‐ethanolamine) and cholesterol, showing the importance of the geometry of those lipids for efficient fusion. In a separate study involving DNA conjugated to 1,2‐O‐dioctadecyl‐rac‐glycerol at either the 3′ or 5′ end, it was shown that both lipid and content mixing of the vesicles took place, indicating vesicle fusion.^74^ The fusion kinetics depended on the DNA sequence and the average number of lipid‐DNA per vesicle. Notably, vesicles without lipid‐DNA or ones presenting noncomplementary sequences underwent lipid mixing or exchange of membrane molecules, but no content mixing. To test the effect of membrane‐membrane spacing on fusion, a series of amphiphilic conjugates was synthesized by adding 2–24 noncomplementary nucleotides at the membrane‐proximal ends of the two complementary sequences. It was found that increasing the lengths of the linkers reduced lipid and content mixing, but increased vesicle docking rates.^75^ To further improve vesicle fusion, we employed DNA modified with four hydrophobic chains, which resulted in stable incorporation of DNA into the liposomal bilayer with limited dissociation, which allowed for an efficient full fusion of the two liposome populations with complementary sequences.^76^

Increased affinity of the hydrophobic domain of the DNA amphiphiles or stronger mechanical coupling between the anchor and the oligonucleotides may improve fusion further. In a striking example of the application of vesicle fusion between an artificial pathogen and a protocell, as shown in Figure 4D, DNA templated docking and subsequent fusion induced by the oppositely charged membranes resulted in gene delivery.^77^ Another excellent example of DNA‐programmed membrane fusion deals with efficient intracellular protein delivery on both suspended and adherent cells.^78^ Thereby, DNA hybridization provides targeting and spatiotemporal control of the fusion between protein‐loaded liposomes and cell membranes, resulting in fast release of proteins into the cytoplasm.

Docking of vesicles in the absence of fusion may lead to vesicle assemblies, which can be controlled by the design of the amphiphilic oligonucleotides. This assembly process, to some extent, is similar to the assembly of DNA‐inorganic nano‐particle conjugates, which was initiated in the 1990s by Mirkin et al.^79^ In contrast to DNA‐covered inorganic nanoparticles, the assembly of multiple vesicles received much less attention. DNA‐controlled assembly of vesicles in solution and on solid supported membranes has been reported however,^80^ using for example a lipid‐DNA conjugate in which ssDNA is coupled to two lipid membrane anchors at either end, with both ends inserting into the lipid membrane while the ssDNA protrudes into the solution. Upon treatment with a complementary DNA strand, the increased stiffness of the double‐stranded DNA releases one of the anchors into the solution, which allows binding to another liposome. Further inter‐liposomal membrane anchoring occurs, which leads to aggregation of the vesicles. This process provides sharp and reproducible thermal aggregation‐disaggregation transitions. The authors proposed that this system might be used to detect biologically relevant polynucleotides. Further optimization of the oligonucleotides and hydrophobic anchor parts allowed detection of DNA sequences at nanomolar concentrations and enabled sensitive mismatch discrimination of target sequences.^81^ Next to thermal disaggregation, liposome assemblies were disconnected into the single vesicle state by means of light (Figure 4E)^82^: A self‐complementary ssDNA bearing a terminal switchable azobenzene moiety was anchored on vesicles and hybridization of the DNA induced vesicle aggregation. Upon irradiation with UV light, the azobenzene isomerizes from the *trans* to a less hydrophobic *cis* isomer, decreasing its anchoring efficacy in the lipid membrane. As a result, the assembly of vesicles was destabilized. Hence, several means of control are present to reversibly assemble and disassemble multivesicle architectures aided by DNA.

## 4 Interactions of DNA Amphiphiles and Their Assemblies with Cell Membranes

The cell membrane, or plasma membrane, plays an essential role in separating the cytoplasm from the extracellular environment, and consequently determines the size of a cell and is involved in cell signaling.^83^ The most common components of the plasma membrane are phospholipids. Another major component is cholesterol, which localizes between the phospholipid molecules and regulates membrane stiffness and stability. Other types of lipids such as glycolipids take up a minor fraction, while membrane proteins occupy a significant portion of the surface. The individual phospholipid molecules are in a dynamic state in which they rotate freely around their long axes and diffuse laterally within each leaflet, thus providing cell membrane fluidity. The cell membrane is not a homogeneously mixed lipid bilayer but displays heterogeneity of the spatial arrangement of lipids and proteins. In some cases, even lipid rafts may be formed. They consist of cholesterol, sphingomyelin and tightly packed saturated phospholipids forming a liquid ordered phase, which is more stable and less fluid than the liquid disordered phase constituting the rest of the membrane.^84^

Here we discuss the interaction of DNA amphiphiles with cell membranes, which provide biological applications from diagnostics to biomedicine.

### 4.1 Anchoring DNA Amphiphiles on Cell Membranes

DNA amphiphiles interact with cell membranes by hydrophobic interactions. In model membranes, DNA amphiphiles dissociate from their micellar aggregates and integrate in model membranes spontaneously.^54^ Next to model membranes, DNA micelles have a strong affinity toward the cell membrane. The hydrophobicity of the DNA amphiphile influences the anchoring on cell membranes, as illustrated by a series of oligonucleotides conjugated to alkyl chains with either 12, 18, or 26 carbons, tested in a range of mammalian cell types.^85^ A strong correlation exists between lipid length and the efficiency with which the amphiphiles are incorporated: Nonfunctionalized DNA shows negligible incorporation, while for DNA with C12 and C18 tails an intermediate insertion efficiency is observed and best piercing into cell membranes is detected for C26. Thereby, ssDNA strands conjugated with fatty acid tails are in a dynamic equilibrium with the culture medium, but when hybridized with its complementary ssDNA that is conjugated with a fatty acid as well, the construct remains in the cell membrane.^86^ Due to double anchoring of the duplex, its interaction with membrane lipids is enhanced, hence the construct remained incorporated into the lipid bilayer.

An alternative mechanism for interaction of amphiphilic DNA with cell membranes is through receptor‐mediated ligand binding. In general, two types of receptor‐mediated ligand interactions are involved: Direct and indirect ones. The DNA segment of the amphiphile can be an aptamer, which selectively targets a cell membrane receptor.^12^ Thus, the amphiphilic DNA attaches to the cell membrane directly. Another receptor‐mediated ligand interaction occurs through an indirect pathway: The lipophilic tail of amphiphilic DNA binds to lipoproteins or other proteins, which are subsequently recognized by the corresponding receptors on the cell membrane. For instance, cholesterol conjugated siRNA can be bound to lipoprotein after intravenous injection into mice.^87^ The high binding affinity of lipoprotein to cellular scavenger receptor SR‐BI facilitates the interaction of cholesterol‐siRNA amphiphiles with the cell membrane. Similarly, octadecyl tails of amphiphilic DNA bound with albumin and the resulting aggregate was recognized by cell surface albumin receptors Gp18 and Gp30.^88^

### 4.2 Factors Influencing the Interaction between DNA Amphiphiles and Cell Membranes

When investigating the interaction efficiency of DNA amphiphiles with cell membranes, one key factor is the structure of the DNA in the amphiphile. Amphiphilic DNA with long DNA sequences incorporates slower into the cell membrane than ones with short nucleic acid chains, because longer DNA forms large micelles with a more densely charged corona, which reduces the availability of the hydrophobic domain.^54^ Another possibility is that longer oligonucleotides contain more anionic phosphate groups, which are repelled by the anionic glycocalyx on cell surfaces.^85^ Next to this, the hydrophobic tail of DNA amphiphiles influences the interaction: Diacyllipids DNA have a high affinity for insertion into the cell membrane, single chain C18 lipid DNA shows modest incorporation, while cholesterol modified DNA exhibits the lowest affinity.^43^ Related to these experiments, a single acyl chain DNA‐mediated membrane anchoring is insufficient to mediate cell–cell adhesion, but the cell–cell interaction is achieved when diacyllipids are used.^89^ Moreover, different lipid tails show preference for various lipid domains: In liposome membranes, diacyllipids mainly anchor to liquid or liquid‐ordered domains, while tocopherols anchor exclusively to liquid‐disordered domains. Cholesterols incorporate into membranes depending on the lipid composition of the membrane. Thus, DNA amphiphiles show preference for lipid domains on cell membranes.^84^ Due to the fact that the lipid composition varies with cell type, different lipid tails can direct amphiphilic DNA to different cell types.^87^

The interaction between amphiphile and cell membrane also depends on the amount of amphiphilic DNA. The number of amphiphilic DNA molecules that can be anchored to the cell membrane depends on the initial concentration of DNA amphiphile in the culture medium.^12^, ^85^ A higher starting concentration leads to a higher density of DNA tethering. For interactions driven by aptamer recognition, densely packed aptamers on an amphiphilic micelle induce a multivalent effect, which leads to higher affinity for the cellular membranes.^10^

Moreover, the cell culture medium influences the interaction of DNA amphiphiles with the membrane. The culture medium affects the anchoring efficiency in the decreasing order: PBS > DMEM > PBS with 10% FBS (Fetal Bovine Serum) > DMEM with 10% FBS.^85^ The components in the cell culture medium alter the interaction between amphiphilic DNA and cell membranes. For instance, albumin in albumin‐rich culture medium binds the lipid domain and forms a complex that prevents amphiphilic DNA inserting into the cell membrane.^88^ Hence, cell membranes display additional features that influence their interaction with DNA amphiphiles. It is important to consider their more complex structure compared to model membranes when applying DNA amphiphiles in living systems.

### 4.3 Stability of the Complex between DNA Amphiphiles and Cell Membranes

After binding to the cell membrane, DNA amphiphiles or their assemblies are not static in space and time. Instead, they are in a dynamic exchange with the medium, they can be degraded and they can be subjected to endocytosis.

All DNA amphiphiles are in equilibrium between the aqueous medium and the cell surface. Even though a DNA sequence is connected to a long lipid tail, like C26, it still displays characteristic re‐equilibration. A gradual loss of lipid DNA on the membrane occurs when replacing the cell culture medium.^85^ This loss is a result of adjusting a new equilibrium between DNA amphiphile on the cell membrane and the culture medium. DNA conjugated to an alkyl chain showed a gradual decay on the cell surface.^90^ After the first hour of incubation, only < 20% loss was observed. However, after 2.5 h only 50% of the initial amount of DNA was present on the cell surface. When incubated for 24 h, only a very weak signal originating from the DNA remained. This gradual decay is temperature‐dependent^89^: Surface anchored DNA decayed to 86% of its initial concentration after 160 min at 25 °C, while 67% of its initial concentration was left after the same time period at 37 °C.

Amphiphilic DNA anchors to the outer leaflet of the cell membrane and is subjected to slow endocytosis. C18 and cholesterol modified oligonucleotides are taken up by cells via an energy‐dependent mechanism rather than by passive diffusion.^39^ Indeed, some of the DNA amphiphiles enter cells via endocytosis, while the majority possibly flips and translocates from the cell surface to the organelles during membrane recycling. As micelles, amphiphilic DNA locates initially close to the cell membrane, then disassembles and fuses with the cell membrane.^91^ This cellular uptake of a DNA amphiphile micelle represents a similar uptake mechanism compared to other amphiphilic molecules.^54^ For interactions driven by receptor binding, endocytosis is suggested as the subsequent step after binding of the amphiphilic DNA or amphiphilic DNA embedded lipoprotein with the receptors. These complexes are recognized by corresponding receptors on the cell membrane and subsequently enter cells via receptor‐mediated endocytosis.^92^

### 4.4 Characteristics of DNA Amphiphiles Interacting with Cell Membranes

Amphiphilic DNA and its assemblies interact efficiently with cell membranes and hence offer a facile strategy for further manipulating the cell surface. A major characteristic is that amphiphilic DNA allows convenient cell surface modification. Other common strategies for presenting DNA at cell surfaces, such as expression of a DNA binding domain of a protein at the cell surface,^93^ covalent attachment of DNA to functional groups at the membrane,^94^, ^95^ or a DNA aptamer that binds membrane target sites,^96^ either involve complicated stepwise processes or can only be applied to very limited membrane targets. Instead, employing amphiphilic DNA to modify a cell surface is simple and quick. Coincubating amphiphilic DNA with cells allows spontaneous insertion of the amphiphiles into the cell membrane. This process is fast and can be performed within only 3 min.^90^ Moreover, amphiphilic DNA can be anchored to different cell types, including natural killer cells,^43^ T cells,^12^ and cancerous cells.^11^ This quick modification procedure results in stable anchoring of the DNA in the membrane: The majority of diacyllipid‐DNA locates on the outer leaflet and remains even after 2 h incubation with cells at 37°.^89^ The easily accessible DNA on the membrane is a highly versatile technology platform in vitro and in vivo. To target cell membranes in vivo DNA amphiphiles can be administered locally. DNA amphiphiles injected into mice remained 72 h at the injection site, which reduced to 4 h with DNA that does not contain a hydrophobic tail.^43^ More important, compared with nucleic acids coated on nanoparticles,^97^ modifying cell membrane by amphiphilic DNA insertion is noninvasive and does not involve inorganic components.

## 5 Applications

Hydrophobic domains within nucleic acids allow their easy incorporation into lipid bilayers and facilitate their uptake by living cells. Here we will discuss the biomedical functions of amphiphilic DNA structures, which can be derived from this behavior.

### 5.1 Drug Delivery

Both amphiphilic micelle and liposome nanostructures can be exploited for drug delivery. Until now, a number of examples have been reported demonstrating highly efficient drug delivery with DNA amphiphiles and their assemblies in vitro and in vivo.^12^, ^28^, ^30^, ^47^ Our group loaded the hydrophobic anticancer drug (Doxorubicin) into the interior of DNA‐*b*‐PPO micelles,^15^ which were taken up through receptor‐mediated endocytosis and significantly inhibited growth of Caco‐2 cancer cells. The cellular uptake of the micelles strongly depended on the density of the recognition elements, i.e., folic acid, on the micellar surface. Moreover, DNA amphiphile micelles were very well suited for loading another hydrophobic anticancer drug: Paclitaxel.^39^

**Figure 5:**
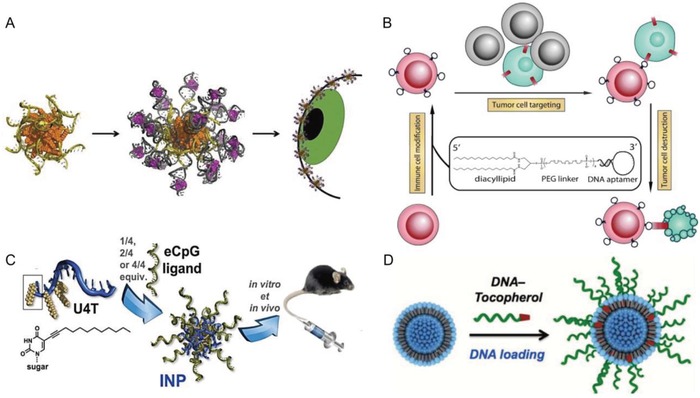
A) Schematic of DNA micelles for treating eye infections. Lipid‐modified DNA strands form micelles and were then hybridized with antibiotics loaded aptamers. Upon administration to the eye, DNA micelles adhere to the cornea and release antibiotics to treat infections. Reproduced with permission.^16^ Copyright 2018, Elsevier. B) Illustration of targeting cancer cells (green) with aptamer‐modified immune cells (red). Immune cells were equipped with lipid modified DNA aptamer, which targets the cancer cell surface. When cancer cells are mixed with normal cells, immune cells only recognize cancer cells by their surface anchored aptamer and then kill cancer cells. Reproduced from ref. 12. C) Schematic of lipid DNA micelles as CpG carrier. Lipid conjugated DNA forms micelle and was then hybridized with different amounts of CpG sequences to form immunostimulatory nanoparticles (INPs) with different degree of CpG functionalization. These INPs activated immune responses both in vitro and in vivo. Reproduced with permission.^98^ Copyright 2018, Elsevier. D) Assembly of liposomal spherical nucleic acids by anchoring tocopherol modified DNA to DOPC small unilamellar vesicles. Reproduced with permission.^22^ Copyright 2014, American Chemical Society.

Recently, we tackled in vivo functionality of the DNA amphiphiles micelle even with human tissue in the context of ophthalmology for treating eye infections.^16^ Therefore, different antibiotics were loaded into the DNA amphiphile micelles (**Figure** **5**A). Aptamers were complexed with an aminoglycoside, i.e., DNA aptamer for Kanamycin B or RNA aptamer for Neomycin B, and subsequently conjugated at the 3′ end of the DNA amphiphiles through hybridization. Compared with pristine drugs, the DNA amphiphile micelles showed extended residence time on the ocular surface and improved efficiency on the cornea in vitro and in vivo. This study highlights the potential applicability of amphiphilic DNA‐based materials in the clinic.

Most recently, a lipid‐conjugated drug‐incorporated oligonucleotide was developed for hitchhiking with endogenous serum albumin for cancer chemotherapy.^88^ By incorporating a hydrophobic lipid tail, floxuridine homomeric oligonucleotides inserted into the hydrophobic pocket of albumin to form complexes which accumulate at the tumor site by the enhanced permeability and retention (EPR) effect and internalize into the lysosomes of cancer cells after intravenous injection. Upon enzymatic degradation, the cytotoxic floxuridine monophosphate is released and inhibited cancer cell proliferation.

### 5.2 Immunotherapy

Furthermore, DNA amphiphiles and their self‐assembled structures find application in immunotherapy. Surface anchoring of DNA amphiphiles directed immune cells to their target cells^12^: Modification of natural killer (NK) cells with an aptamer named KK1B10 (Figure 5B) resulted in specific targeting of cancer cells, i.e., chronic myelogenous leukemia cell line K562. This resulted in 50% increased killing efficiency of NK cells toward K562 cancer cells, compared with unmodified NK cells. The higher killing efficiency was likely due to the better targeting efficiency of NK cells when the DNA aptamer amphiphile is attached. Moreover, the selectivity of the aptamer modified NK cells was demonstrated when the target K562 cells are mixed with an excess of nontargeted cells.

In a different approach, the immunological effects of DNA amphiphile micelles decorated with the immune adjuvant (CpG) were studied in vivo recently.^98^ Different amounts of immunostimulatory adjuvants were established on the surface of spherical micelles through simple stoichiometric incorporation (Figure 5C). After that, a full immunological assay, including phagocytosis, the expression of costimulatory molecules, and the production of proinflammatory cytokines in spleen dendritic cells (DCs) was evaluated and analyzed. As a result, dose‐dependent activation of spleen DCs by CpG‐conjugated micelles was observed, which was accompanied by the pronounced up‐regulation of costimulatory molecule and cytokine production. In addition, labeling 50% of the DNA amphiphile micelles with the CpG segment can fully induce the activation of spleen DC. The straightforward functionalization by DNA duplex formation makes the DNA amphiphile micelles a biocompatible and scalable delivery platform for immunostimulation and immunotherapy. Since such DNA micelles still exhibit single‐stranded DNA on the surface ready for hybridization, these sites could be easily exploited for the incorporation of antigens to boost the generation of humoral and cellular vaccine‐specific immune responses.

### 5.3 Gene Silencing

Gene silencing offers the potential to cure certain diseases by down‐regulating the disease‐causing gene expression and protein production.^8^ One of the most widely used gene silencing strategies is exogenously derived single‐stranded antisense oligonucleotides (ASOs). As discussed in the introduction part, the intrinsic physicochemical properties of ASOs, such as negative charges, high hydrophilicity, and high molecular weight, prevent their efficient delivery to the intracellular target site.^99^ To this end, conjugation of hydrophobic moieties to ASOs has been used as a safer and straightforward strategy to assist their cellular uptake.^100^, ^101^, ^102^ Early studies from the 1980s used cholesteryl conjugated oligonucleotides to inhibit HIV infections^103^, ^104^ or targeted the intercellular adhesion molecule‐1 gene.^105^ Later, hydrocarbon lipids were conjugated to oligonucleotides to assist antisense efficiency: Barthélémy et al. proposed an example involving lipid moieties that were connected to oligonucleotides via click chemistry, which promoted cellular uptake.^39^ As a result, the hepatitis C virus (HCV) internal ribosome entry site (IRES)‐mediated translation was effectively suppressed. Interestingly, when the ASO was conjugated to a C18 lipid or cholesterol unit, a dose‐dependent reduction of the translation was measured in the Huh7 cell line. More importantly, the biological activity of the oligonucleotide was not affected by the lipid conjugation and toxicity was negligible at relevant concentrations. In another notable example, Mirkin and coworkers synthesized a spherical nucleic acid nanostructure, which consists of a liposomal core (30 nm) stabilized with a dense shell of tocopherol‐modified DNA that intercalates between the phospholipids and defines the liposomal structure (Figure 5D).^22^ By using commercially available and FDA‐approved building blocks, they demonstrated that such monodisperse DNA‐functionalized vesicles remain stable with no change in dispersity for at least 4 days at 37 °C. This behavior is contrary to native nonfunctionalized vesicles, which tend to fuse and form large poly‐disperse structures under such conditions. The obtained spherical nucleic acid architecture did not only stabilize the liposomal constructs but rapidly entered multiple cell lines and resulted in effective gene knockdown of HER2 in SKOV‐3 cells.

### 5.4 Sensing the Extra and Intracellular Environment

Tracking cell functions, metabolism, and cell–cell signaling in their native cellular environment has enormous implications for cell biology and regenerative medicine.^106^ For the past few decades, molecular sensors^107^, ^108^ or nanoparticles^109^ tethered on the membrane surface have been utilized to monitor such cell activities. However, these sensors exhibit several drawbacks, such as limited targets, a need for complicated chemical modification, allowing measurements only under model conditions, or they do not monitor in real‐time.^106^ Fortunately, as a relatively new cell surface biosensor, amphiphilic DNA outperforms other methods in several aspects. First, aptamers can be selected via a process called systematic evolution of ligands by exponential enrichment (SELEX) to specifically bind to certain target molecules, such as metal ions, small organic molecules or proteins with high affinity. Second, the straightforward functionalization of DNA with fluorophores facilitates signal readout by means of photoluminescence. Furthermore, hydrophobic tags permit anchoring of the biosensor to the cell membrane. Finally and importantly, DNA hybridization or the fast response of DNA aptamers for their targets render monitoring in real time and in situ with high spatiotemporal resolution feasible. However, sometimes the action of aptamers is compromised by nuclease degradation, variability of pharmacokinetics or rapid renal filtration in native environments.^110^ To overcome these limitations, their activity or persistence under physiological conditions were optimized during selection.^3^ Another means of stabilization represents the introduction of chemical modifications to decrease enzymatic digestion, and PEGylation to prolong circulation times.^111^

**Figure 6:**
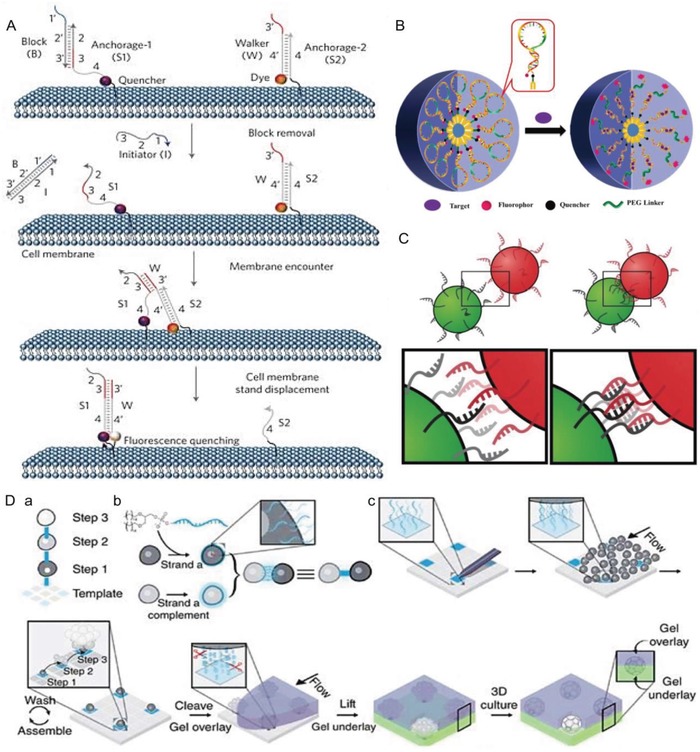
A) Schematic illustration of using a DNA probe to measure the encounter rate of lipid domains on live cell membranes. The S1 strand is anchored to the cell membrane and is partially hybridized with a blocking strand B. Similarly, the S2 strand is anchored at a second anchor site and partially hybridized with a walker strand W. When an initiator stand I is introduced to the system, it removes strand B from S1, leaving S1 free for hybridization. Since the walker strand from the S2 site has priority to hybridize with this free S1 strand over its own S2 strand, it will translocate from the S2 site to the S1 site once both strands meet each other. Since the S1 and S2 stands are labeled with FRET dyes, once they encounter each other, the fluorescence is quenched. Different hydrophobic moieties attached the nucleic acid units introduce selectivity of the DNA strands for certain lipid domains. Thus the quenching rate is a measure to evaluate the encounter rate of different lipid domains. Reproduced with permission.^40^ Copyright 2017, Nature Publishing Group. B) Working principle of switchable aptamer micelle flares for ATP imaging inside living cells. On the left, aptamers are folded, while upon binding the target molecule, the aptamer unfolds leading to a dequenching of fluorescence (right side). Reproduced with permission.^91^ Copyright 2013, American Chemical Society. C) Two populations of cells exhibit anchored DNA on their membranes. When mixed together, hybridization of membrane‐embedded DNA induces cell–cell contact. Reproduced with permission.^114^ Copyright 2009, National Academy of Sciences. D) Illustration of microtissues constructed by DNA hybridization. a) Illustration of cell adherence by DNA hybridization. One type of cell is anchored with a particular DNA strand on the membrane. The other cell type is functionalized with the complementary DNA strand. b) When the two cell types are mixed, hybridization induces cell‐to‐cell aggregation. c) Formation of microtissue by DNA hybridization. Iteration of this process allows assembling microtissues into the third dimension. Reproduced with permission.^115^ Copyright 2015, Nature Publishing Group.

So far, amphiphilic DNA has been used to monitor metal ions,^112^ pH^113^ and chemical transmitters^41^ in cellular environment. Another notable example is the measurement of formation of lipid membrane domains to monitor and understand the dynamic signaling interactions on the cell surface (**Figure** **6**A).^40^ To achieve this, a ssDNA strand named S1 was anchored to the cell membrane via a hydrophobic lipid unit and was partially hybridized with a blocking strand B. Similarly, a S2 strand was anchored at a second anchor site and was partially hybridized with a walker strand W. An initiator strand can completely remove the blocking strand from the S1 strand by a strand displacement reaction, leaving S1 free for hybridization. Because strand W from the S2 site hybridizes preferentially with the free S1 strand, it will translocate once both sequences are in close proximity. To observe this displacement, strand S1 and strand W were labeled with a fluorescence resonance energy transfer (FRET) pair, leading to quenched fluorescence. The FRET efficiency becomes a measure of the lipid domain encounter rate since the DNA amphiphiles were anchored in different lipid domains. Three lipid tails were attached to the nucleic acid moie‐ties, i.e., diacyllipid, cholesterol, and tocopherol, to specifically locate DNA strands in different cellular lipid domains. This method transduces transient encounters of nanodomains into a cumulative cell surface fluorescence signal and thus allows to detect signaling events on live cell membranes.

Apart from probing the cell surface, amphiphilic DNA was utilized for imaging and detecting intracellular parameters such as the level of ATP.^91^ A switchable aptamer‐containing micelle flare allowed detection of ATP within cells (Figure 6B). This design implicated three segments with a DNA layer that folds into an aptamer loop against ATP. The hydrophobic segment was a diacyllipid tail, with a PEG unit as spacer between the DNA and the hydrophobic tail. A fluorophore and a quencher were covalently attached to 3′ and 5′ ends. Once ATP is binding, the DNA loop opens, leading to an increase of fluorescence. Due to the fact that the micelles interacted with the cell membrane and were internalized into cells, ATP in both membrane and cytosolic environment could be detected.

### 5.5 Cell Capture and Assembly

Similar as the DNA amphiphile mediated liposome assembly discussed in Section 3.2.2, when tethered onto the cell membrane, the amphiphilic DNA facilitates cell capture and assembly through the specific and fast recognition properties of the nucleic acids. The length of the DNA strands is of crucial importance for the successful cell to cell contact by hybridization.^89^ A 20‐mer DNA strand on the cell surface cannot hybridize with its complementary sequence due to steric hindrance provided by the dense glycocalyx layer. However, a 60‐ and 80‐mer poly(dT) spacer inserted between the lipid anchor and the DNA recognition element that will hybridize, significantly increases the cell adhesion to other surfaces. Eventually, DNA‐anchored on cell surfaces can be linked to surface‐anchored complementary DNA.

The accessibility of cell membranes with anchored DNA amphiphiles also facilitates cell assembly and microtissue formation. In one example, Bertozzi et al. linked nonadherent Jurkat cells together by employing DNA anchored on their surfaces (Figure 6C).^114^ This group found that the most important parameters for cell assembly are the cell concentration, DNA density on the cell surface, and DNA sequence complexity. Since the cells are attached to each other through DNA hybridization, this process can be reversed by DNase addition or thermal melting. This allows the construction of microtissues with defined cell composition and stoichiometry. This approach can be extended in a bottom‐up strategy that uses a DNA‐patterned substrate as a template and temporary DNA‐based cellular adhesions as synthetic linkages between cellular building blocks for tissue engineering in 3D (Figure 6D).^115^ In this way, the construction of arrays of 3D cell cultures with many tunable parameters was feasible. In the same study, template DNA was linked to a glass slide to form DNA patterns. Then, a PDMS flow channel was placed on top of the DNA pattern. A cell population functionalized on the surface with complementary DNA to the template DNA was added to the flow channel, which directed the cells to the designed 2D pattern. The formed cell pattern could be released by enzymatic cleavage of the DNA. Embedding such microtissues constructed from DNA in gels allows to study the influence of tissue size, shape and composition on cell behaviors in 3D.

### 5.6 Complex DNA Nanostructures on and in the Cell Membrane

**Figure 7:**
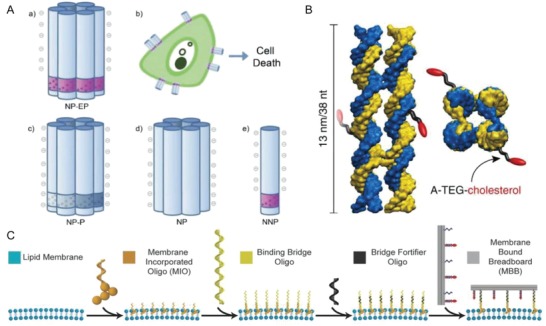
A) A membrane‐spanning DNA nanopore (NP) with cytotoxic activity and three negative control nanostructures. a) The NP‐EP pore is composed of a six‐duplex bundle (blue) and a hydrophobic belt (purple) made up of 72 ethyl phosphorothioate (EP) groups. b) Inserting of NP‐EP pores into cellular membrane induces cell death. c) NP‐P features phosphorothioate groups but no hydrophobic ethyl modification. d) NP contains native phosphate groups. e) NNP contains EP groups but lacks three of the six strands required to generate the six‐duplex bundle nanopore. Reproduced from ref. 122. B) Design of the lipid‐scrambling DNA nanostructure. Reproduced with permission.^123^ Copyright 2018, Nature Publishing Group. C) Illustration of the sequential MBB functionalization steps. Oligos (MIO) are first integrated into the cell membrane, then bridge oligos hybridize with MIO strands followed by bridge fortifier oligo hybridization. Lastly, membrane bound breadboard (MBB) binds to the cell membrane by hybridizing with bridge oligos. Reproduced from ref. 124.

The extraordinary self‐recognition and hybridization properties of DNA can be applied for creating various programmable nanostructures.^116^ An exceptional form of DNA amphiphiles are DNA‐based nanopores. DNA‐based nanopores open exciting opportunities in the field of bio‐nanotechnology, as shown by their protein‐based counterparts.^117^ Single‐stranded nucleic acid scaffolds together with staple strands or short oligonucleotides can fold into DNA‐based nanopores. When conjugated to hydrophobic units, the otherwise hydrophilic nanopores insert into synthetic lipid membranes.^118^, ^119^, ^120^, ^121^ Moreover, these nanopores interact with biological membranes (**Figure** **7**A).^122^ A notable example is a DNA nanopore with a 2 nm opening and an outer diameter of 5.5 nm and a height of 14 nm, which contained a hydrophobic belt with 72 ethyl phosphorothioates at the bottom of the pore to direct insertion into the cell membrane. After incubating these nanostructures with cervical cancer cells, the DNA nanopores mainly located at the membrane and caused cell death. Nanopores that did not contain a hydrophobic belt were mostly internalized by the cancer cells. The cytotoxic effect of DNA‐based nanopores could allow for anticancer activity, albeit for true applications the selectivity needs to be improved.

Apart from a cytotoxic effect, DNA nanostructures on cell membranes enable the transport of membrane lipids. A lipid‐scrambling DNA nanostructure, consisting of only eight DNA strands, which were modified by tetraethylenglycol (TEG)‐cholesterol (Figure 7B),^123^ spontaneously inserts into biological membranes by forming a toroidal pore that connects the inner and outer leaflets of the membrane. The inserted nanostructure facilitates the exchange of lipid molecules between the inner and outer bilayer leaflets rapidly equilibrating the lipid composition. The rate of lipid transport catalyzed by the DNA nanostructure is three orders of magnitude higher than that reported for lipid transport catalyzed by natural enzymes. The stable DNA‐induced toroidal lipid pore likely induces this exceptional transport behavior. The DNA‐based artificial scramblase also showed translocation of phosphatidylserine lipids from the inner membrane leaflet to the outer leaflet of human cancer cells.

Besides insertion, DNA‐origami nanodevices can be placed onto the surface of living cells (Figure 7C).^124^ The membrane can be functionalized by anchoring DNA to the cell surface via cholesterol insertion into the membrane, followed by binding of a bridge‐oligonucleotide that partially hybridizes with this surface DNA. The bridging oligo allows binding of the membrane‐bound breadboard (MBB) binding sites, but also offers the possibility of removal of this MBB from another surface via a strand displacement reaction. Several cell types can be functionalized with MBBs, including primary, endothelial, and lymphoma cells. Furthermore, the MBB can be released from cell surfaces when a detachment strand is added. By using DNA origami nanodevices as engineering tools, MBB acts as a mediator for either homotypic or heterotypic cell–cell interactions, which mimic complex biological processes on the cell membrane.

## 6 Conclusions and Perspective

DNA‐based materials have exceptional properties in regard to structural design. Compared to other building blocks like peptides, proteins, and synthetic macromolecules, DNA allows the bottom‐up construction of complex architectures and tuning the interaction energy between complementary DNA strands. Recent progress in the design and functionalities of DNA amphiphiles builds on these remarkable properties to implement DNA hybrid materials into the application areas of diagnostics and biomedicine. These efforts are enabled by well‐established protocols to synthesize amphiphilic DNA molecules and their commercial availability. Moreover, the topology and interactions of amphiphilic DNA is highly controllable, and their aggregation behavior into superstructures such as micelles or vesicles, but also many other geometries can be precisely adjusted. It is possible to tune their size, switch their assembly state, and modify their surfaces at will through duplex formation. With their hydrophobic units, amphiphilic DNA hybrids further provide a simple and efficient strategy for membrane modification of living cells. This simple functionalization procedure allows further cell surface engineering, cell assembly, and facilitates potential sensing applications.

Despite these many favorable properties of DNA amphiphiles, certain challenges need to be overcome before translating them further toward the clinic. One of the most critical issues is the biological stability. Although enhanced enzymatic stability was reported for DNA amphiphile micelles,^64^ it remains a challenge to minimize nuclease degradation, especially in vivo. Next to this, upon exposure to biological medium, amphiphilic DNA structures are encapsulated by a protein corona,^125^ which possibly shields recognition elements on the surface and compromises its targeting efficiency. Another big challenge represents maintaining the solubility of amphiphilic DNA in biological media and its activity on membranes. Proteins from serum, like albumin or lipoproteins, are well known to form stable complexes with amphiphilic DNA,^126^, ^127^ thus preventing their desired functions. Approaches to prevent such interactions of amphiphilic DNA with serum proteins are urgently needed for extending biomedical applications.^128^ Furthermore, amphiphilic DNA molecules in micelle assemblies are always in a dynamic equilibrium within their environment: Strong dilution after intravenous injection might result in concentrations below the CMC, which leads to disassembly of micelles and drug release before reaching the target.^129^, ^130^ To prevent this, the biological stability of amphiphilic DNA micelle needs to be adjusted to the desired delivery function. It has been shown that covalent cross‐linking of the lipid DNA molecules can for example increase the stability of the assembled nanostructures,^65^ and therefore we foresee that this challenge will be overcome in the near future.

Although the introduction of a hydrophobic segment into the nucleic acid amphiphiles is essential for their function, the biocompatibility and biosafety of these hybrids should be taken into consideration, especially toward clinical translation. For example, too many lipid‐DNA insertions in the membrane will lead to cell membrane disturbance, damage, and cell death.^12^, ^85^ Since the insertion mechanism into membranes, which is mediated by hydrophobic interactions, is not specific for a given cell type, it is essential that additional features for selective incorporation are introduced. A notable example of such an effort is labeling the DNA amphiphiles with folic acid to target cancer cells.^15^ At this stage, most studies on the biocompatibility of DNA amphiphiles were conducted using cell cytotoxicity evaluation, while only a few studies investigated their local or systematic toxicity in vivo.^16^, ^43^ As more and more DNA amphiphiles are developed for biomedical applications, these activities need to be extended for more comprehensive toxicological evaluations, such as cell membrane damage, cell signaling interference, oxidative stress, genotoxicity, etc., which are required for predicting long‐term biosafety.

Since a lot of knowledge and control over synthesis and assembly mechanisms of DNA amphiphiles have been gained, we are in an excellent position to explore the unique properties of DNA amphiphiles when combined with hydrophobic molecules. Similar as native protein clusters on cell membranes, DNA nanostructures (not limited to nanopores) might act as artificial gate for intracellular/extracellular transportation, as means for cellular environment regulation and as tool to regulate cellular signaling. From the perspective of synthetic biology, the exciting examples of interfacing DNA amphiphiles with membranes will fuel further activities regarding artificial cell engineering, cell assembly and novel tissue formation.^131^ Moreover, DNA amphiphiles might find potential applications in cell‐based therapy. In addition to immune cells, amphiphilic DNA nanostructure hitchhiked on other circulatory cells merits more investigations.^132^

Taken together, DNA amphiphiles are at a stage where a large variety of nucleic acid materials is readily available, hence several structural designs were investigated in combination with living sytsems, especially addressing potential biomedical applications. We predict a further growth in this area addressing more complex functions including the fields of oncology, vaccination and theranostics.

## Conflict of Interest

The authors declare no conflict of interest.
